# Efficacy and Safety of Bupivacaine Liposomal in Intercostal Nerve Block for Postoperative Pain Management Following Uniportal Thoracoscopy: A Randomized Trial

**DOI:** 10.1155/prm/8816879

**Published:** 2025-07-09

**Authors:** Lingjun Dong, Xiang Wang, Linhai Fu, Zongming Jiang, Yulong Wang, Aixia Chen, Jianyi Ding, Guangmao Yu

**Affiliations:** ^1^Department of Thoracic Surgery, Shaoxing People's Hospital, 568-Zhongxing North Road, Shaoxing 312000, Zhejiang, China; ^2^Department of Anesthesiology, Shaoxing People's Hospital, 568-Zhongxing North Road, Shaoxing 312000, Zhejiang, China; ^3^Department of Nursing, Shaoxing People's Hospital, 568-Zhongxing North Road, Shaoxing 312000, Zhejiang, China

**Keywords:** intercostal nerve block, liposomal bupivacaine, multimodal analgesia management, randomized controlled clinical trial, uniportal thoracoscopic surgery

## Abstract

**Background:** Postoperative pain in thoracic surgery often requires opioids, yet can be poorly managed with short-acting anesthetics. Liposomal bupivacaine (LB) offers prolonged analgesia, potentially improving pain control and reducing opioid use. This study evaluates LB's effectiveness and safety in thoracic postoperative pain management, aiming to provide an alternative to current practices.

**Methods:** In this single-center, double-blind, prospective, randomized controlled trial, patients undergoing uniportal lobectomy, segmentectomy, or wedge resection from November 2023 to May 2024 were enrolled. Participants were randomly assigned in a 1:1 ratio to receive either 0.375% ropivacaine (control group, *n* = 57) or LB (LB group, *n* = 56) for intercostal nerve blocks (ICNBs). Postoperative visual analog scale (VAS) scores, opioid consumption, overall benefit of analgesia score (OBAS), chest tube duration, length of hospital stay, and adverse events (AEs) were recorded and analyzed.

**Results:** Data from 57 patients in the control group and 56 patients in the LB group were included in the analysis, with no significant demographic differences between the groups. The LB group demonstrated lower VAS scores at rest and during activity (*p* > 0.05), reduced opioid consumption (*p*=0.021), and higher OBAS (*p* < 0.01) compared with the control group. No significant differences were observed in chest tube duration, length of hospital stay, or AEs between the groups.

**Conclusion:** LB is safe and effective for ICNB, providing significant postoperative pain relief for patients undergoing uniportal thoracoscopic surgery.

**Trial Registration:** Chinese Registry of Clinical Trials: chiCTR2300075463

## 1. Introduction

Postoperative pain management in thoracic surgery has gained significant focus in recent years due to the frequent occurrence of moderate to severe pain [1]. Traditionally, opioids have been the cornerstone for managing postoperative pain in thoracic surgery. However, advancements in pain management strategies have highlighted the superior analgesic effects of regional nerve block techniques over conventional intravenous methods, significantly reducing opioid-related side effects [2].

Intercostal nerve block (ICNB) is favored among common regional nerve block techniques due to its simplicity and safety. It does not require additional specialized catheter equipment and does not prolong anesthesia or surgery time. However, the effectiveness of existing local anesthetics for ICNB typically lasts only 12–18 h, leading to breakthrough pain and increased pain intensity scores [3]. To extend the duration of regional anesthesia, adjuvants in single-shot techniques or continuous catheter techniques are employed [3, 4]. Nevertheless, continuous catheter placement poses several challenges, including risks of infection, dislodgement, migration, leakage, and difficulties in removal [5–7].

Liposomal bupivacaine (LB) offers a significant advancement in this field by utilizing multivesicular liposome technology to extend the release of bupivacaine up to 72 h, far exceeding the duration of water-soluble local anesthetics [8]. International guidelines have started to include LB in multimodal analgesia protocols to support faster patient recovery [9–11]. Despite this, research on the analgesic efficacy of LB combined with systemic analgesia for ICNB in thoracic postoperative pain management remains limited. Therefore, our team is conducting a prospective, single-center, double-blind, randomized controlled trial to evaluate the efficacy and safety of LB for postoperative pain management in thoracic surgery.

## 2. Materials and Methods

### 2.1. Study Overview

This is a single-center, double-blind, prospective, randomized controlled clinical trial. The study follows the Consolidated Standards of Reporting Trials (CONSORT) guidelines and received approval from the Academic Ethics Committee of Shaoxing People's Hospital (Document no.: IEC-K-AF-016-1.2) in 2023. Informed consent was obtained from all participants prior to their involvement in the study.

### 2.2. Patients

Patients eligible for this study are those aged 18 and older, willing to provide informed consent, and scheduled to undergo uniportal video-assisted thoracoscopic surgery (VATS) between November 2023 and May 2024, including lung lobectomy, segmentectomy, or wedge resection. Inclusion criteria and exclusion criteria are detailed in the Supporting Information (available here).

### 2.3. Study Design

Patients are randomly assigned in a 1:1 ratio using a randomization table to either the LB group or the control group. All patients receive ICNB, with the LB group receiving LB (266 mg/20 mL) and the control group receiving 0.375% ropivacaine (75 mg/20 mL) prepared by diluting 7.5 mL of 1% ropivacaine with saline to 20 mL. Due to the distinct appearance of LB solution (a white to off-white milky suspension) and ropivacaine hydrochloride (a clear colorless liquid), blinding of dispensing and administering personnel is not feasible. An independent assessor is designated for the trial. Drug preparation and administration are performed by nonblinded personnel, while patients and caregivers (blind personnel) are required to avoid contact with the study drug.

### 2.4. Anesthesia Protocol

The anesthesia protocol and postoperative pain management strategy, including induction, maintenance, monitoring, patient-controlled intravenous analgesia (PCIA), surgical technique, ICNB, and postoperative pain management, are detailed in the Supporting Information.

### 2.5. Study Endpoints

#### 2.5.1. Primary Study Endpoint

The primary endpoint is the effectiveness of postoperative analgesia, assessed by the VAS at multiple time points: 4 h (±10 min), 8 h (±10 min), 12 h (±10 min), 16 h (±15 min), 20 h (±15 min), 24 h (±15 min), 32 h (±30 min), 40 h (±30 min), 48 h (±30 min), 60 h (±30 min), and 72 h (±30 min) after the end of anesthesia. VAS scores are also recorded following patient activities such as coughing or moving out of bed. If the patient is asleep at these time points, they are not awakened, and a VAS score of 2 is recorded. If the chest drain is removed within 72 h, the final VAS is recorded before removal.

#### 2.5.2. Secondary Study Endpoints

(1) Postoperative rescue opioid dose (0–72 h), converted to oral morphine equivalents (OMEs). (2) Frequency of PCIA use (0–72 h). (3) Patient satisfaction with overall analgesia, measured by the overall benefit of analgesia score (OBAS) questionnaire at extubation or at 72 h for those not extubated within 72 h (“How satisfied are you with your pain treatment during the past 24 h?” [0: not at all to 4: very much]) [12]. (4) Occurrence of adverse events (AEs): nausea, vomiting, rash, fever, cardiac arrhythmias, electrolyte disturbances, liver and renal function abnormalities, and other AEs.

### 2.6. Statistical Analysis

Based on literature review and pretest results, the mean 24-h postoperative VAS was 1.4 for the LB group and 1.7 for the control group, with an assumed overall standard deviation of 0.5. With a one-sided *α* = 0.05 and 90% power, a 1:1 test-to-control ratio, and a 20% dropout rate, a total sample size of 120 patients (60 per group) was determined.

Data collected from the medical record system include patient demographics, hypertension, diabetes mellitus, length of tube placement, length of hospitalization, tumor location, tumor size, extent of surgical resection, postoperative rescue opioid use, PCIA use, complications, and AEs. Opioids are converted to OME for analysis.

Statistical analysis is performed using SPSS 27.0 software. Categorical variables are presented as percentages and analyzed using the chi-square test. Normally distributed continuous variables are expressed as the mean ± standard deviation (mean ± SD) and compared using an independent samples *t*-test. Non-normally distributed continuous variables are expressed as the median or interquartile range and analyzed using the Mann–Whitney *U* test. Statistical significance is defined as *p* < 0.05.

## 3. Results

### 3.1. Patient Demographic Characteristics

**Figure 1:**
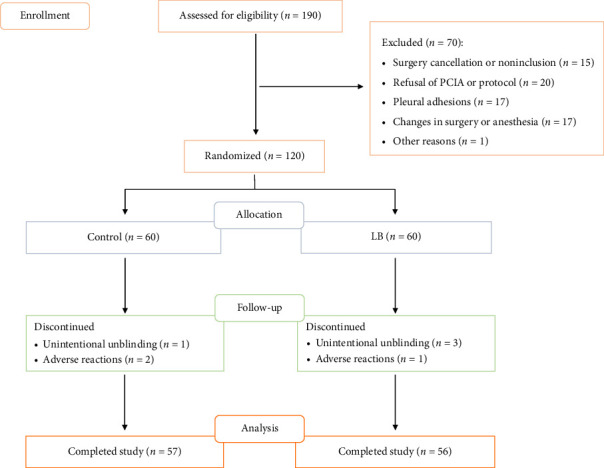
CONSORT diagram of the participants in trial. LB = liposomal bupivacaine.

A total of 190 patients were initially recruited for this study. Exclusions included 15 patients due to surgical cancellation or not meeting inclusion criteria, 20 patients who refused PCIA or its regimen, 17 due to thoracic adhesions, 17 due to changes in surgical or anesthesia plans, and 1 due to other reasons. Ultimately, 120 patients were randomly assigned to the control and LB groups. After observation, 113 patients successfully completed the trial (57 in the control group and 56 in the LB group) and were available for subsequent analysis (Figure 1). Both groups were similar in age, gender, height, weight, BMI, smoking history, alcohol history, hypertension, diabetes mellitus, tumor location, tumor size, and extent of resection. However, the control group had a higher proportion of benign lesions compared with the LB group (17.7% vs. 5.31%, *p*=0.002). Overall, baseline characteristics, such as demographic data, lesion size, location, and extent of surgical resection, were consistent between the groups (Table 1).

### 3.2. Primary Endpoints

**Figure 2:**
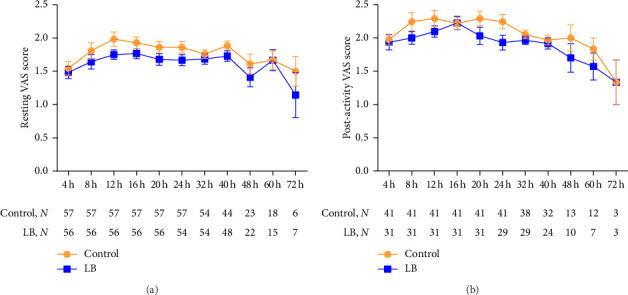
Time trend in postoperative resting and postactivity VAS scores. (a) Postoperative resting, mean ± SE. (b) Postactivity VAS scores, mean ± SE. LB = liposomal bupivacaine; VAS: visual analog scale.

The multimodal analgesic regimen of ICNB combined with PCIA provided effective pain control postminimally invasive thoracic surgery. In the control group, the mean VAS score at rest during the short-term postoperative period (0–72 h) was below 2, with a median of 1.86 (1.5–1.982). The VAS score increased after activity, with a median of 2.053 (1.333–2.293). In the LB group, the VAS scores at rest fluctuated around 1.5, with a median of 1.67 (1.143–1.768), and increased after activity, with a median of 1.935 (1.333–2.226). The VAS scores peaked at 12 h postoperatively and then gradually declined. The LB group had consistently lower postoperative VAS scores compared with the control group at all time points though the differences were not statistically significant (Figures 2(a) and 2(b)).

### 3.3. Secondary Endpoints

No significant difference was found in OBAS between the groups (4 [3, 4] vs. 4 [4], *p*=0.11). However, a higher percentage of patients in the LB group were very satisfied with their pain management (OBAS = 4) compared with the control group (36/57 vs. 47/56, *p*=0.001). The median length of postoperative stay was the same for both groups at 3 [2, 4] days, with no statistical difference (mean postoperative stay was 3.684 vs. 3.214, *p*=0.27). Chest tube duration also did not differ significantly between the groups (45 [40.61.5] vs. 45 [41.56.75], *p*=0.63) (Table 2).

The remaining PCIA volume was higher in the LB group (92 [86.97] vs. 95 [90.98], *p*=0.05), indicating more frequent use of PCIA by the control group (mean residuals 90.39 mL vs. 93.79 mL, *p*=0.02). There was no significant difference in rescue opioid usage between the groups (0 [0.33.3] vs. 0 [0.0], *p*=0.25). However, total opioid consumption was significantly lower in the LB group, with patients using 24 mg less OME compared with the control group (48 [18.93] OME vs. 24 [7.5.65.33] OME, *p*=0.02) (Table 2).

In addition, we assessed cumulative and daily opioid consumption between the two groups.  Table 3 demonstrates a disparity in daily PCIA usage and opioid consumption, with the LB group exhibiting reduced consumption. On postoperative day 1, opioid demand was markedly higher compared with Days 2 and 3. Moreover, the LB group's cumulative PCIA usage and opioid consumption were significantly lower than the control group. Overall, patients receiving LB experienced a 78.57% reduction in total opioid consumption during hospitalization.

### 3.4. Complications and AEs

AEs within 72 h postsurgery were evaluated. The incidence of any AE was 35% (21 cases) in the control group (*n* = 60) and 25% (15 cases) in the LB group (*n* = 60). No serious adverse events (SAEs) were reported in either group. Follow-up was interrupted due to AEs in 2 cases (3.3%) in the control group (one patient with a rash and another with severe nausea and vomiting) compared with 1 case (1.7%) in the LB group (severe low back pain) (Table 4). Overall, the LB group had a lower incidence of AEs compared with the control group, and no SAEs were reported, indicating a favorable safety and tolerability profile.

## 4. Discussion

At our center, over 90% of lung surgeries are performed using uniportal VATS, which offers the benefits of reduced trauma, minimal bleeding, and quicker recovery times [13]. ICNB is the most frequently employed local analgesic technique following thoracic surgery and is effective in managing postoperative pain [14]. Ullah et al. demonstrated that patients receiving ICNB experienced better pain relief within the first 6 h postsurgery and required less morphine compared with those who did not receive ICNB. However, beyond the 6-h mark, there were no significant differences in pain scores or morphine consumption between the two groups [15]. Even with long-acting local anesthetics like ropivacaine, pain control effectiveness diminishes significantly after 6 h and is entirely absent after 2 days, leading to increased postoperative opioid use and related AEs [16, 17]. Multimodal analgesia, which combines drugs with different mechanisms and local blockade techniques, aims to achieve superior pain control, reduce complications, and maintain homeostasis [18]. LB has been shown in several studies to provide analgesia for up to 72 h [19, 20]. Therefore, we conducted a randomized controlled trial to investigate the efficacy and safety of LB for ICNB combined with a PCIA regimen in managing postoperative pain after uniportal thoracoscopy.

In our study, we observed that the multimodal analgesia regimen of ICNB combined with PCIA was highly effective for pain control after uniportal thoracoscopy. In the control group, the mean resting VAS score remained below 1.982, while in the LB group, it was below 1.768 during the first 72 h postoperatively. The mean VAS scores during activity were also low, remaining below 2.293 in the control group and 2.226 in the LB group, indicating effective pain management in both groups. This finding aligns with a retrospective study showing that postoperative pain scores for patients receiving LB for ICNB combined with local incision block averaged below 1.9, with a duration of analgesia comparable to that of thoracic epidural analgesia [21]. Furthermore, our study showed that the VAS curves for both groups started to diverge at 4 h postoperatively, peaked at 12 h, with the LB group consistently showing lower VAS scores. In addition, a higher number of patients in the LB group reported being very satisfied with their pain control, which meets our expectations and underscores the potential of LB in enhancing postoperative analgesia.

Regarding secondary endpoints, our findings indicate that patients receiving LB experienced a 66.7% reduction in the median number of PCIA uses and a 50% reduction in the median amount of opioids used within 72 h. Although rescue opioid use did not show a significant statistical difference between the groups, 16 patients in the control group required a total of 30 instances of rescue analgesia for breakthrough pain, compared with 11 patients in the LB group who required 14 instances. This suggests that LB can effectively reduce the incidence of breakthrough pain and overall opioid consumption. Daily PCIA usage and opioid consumption were significantly lower in the LB group, highlighting the prolonged analgesic effect of LB in postoperative pain management. Consistent with our findings, several retrospective studies have reported that LB for local blocks in thoracic surgery significantly reduces opioid use and frequency [21–23]. Reducing opioid use enhances postoperative patient experience and safety and mitigates issues related to opioid abuse and addiction. To this end, the PCIA background rate was set to 0 mL/h in this trial. Overall, the use of LB in postoperative pain management in thoracic surgery offers substantial benefits.

Previous retrospective studies have also indicated that LB use is associated with shorter hospital stays [21, 23]. However, our study did not find significant differences in the duration of tube placement or postoperative hospital stay between the two groups. The median tube placement duration was 45 h, and the median postoperative hospital stay was 3 days for both groups. This outcome may be influenced by our center's practice of issuing tube removal orders after morning rounds and keeping patients for an additional day of observation post-tube removal before discharge. Consequently, postoperative hospital stays at our center typically exceed tube removal duration by one day. Furthermore, due to anesthesia reactions and personal preferences, patient activity is often limited within the first 24 h postoperatively. Between 24 and 48 h, patients are encouraged to increase activity and cough effectively to expel residual gas and fluid, meeting the criteria for tube removal. Premature tube removal could lead to residual thoracic cavity gas and fluid, necessitating retube placement. For safety reasons, our center generally removes tubes between 24 and 48 h postoperatively, making it challenging to shorten the postoperative hospital stay.

We observed that LB can be safely used in ICNB. The incidence of AEs in the LB group was 25%, lower than the control group's rate, potentially due to reduced postoperative opioid demand among patients receiving LB, which resulted in fewer opioid-related AEs. We noted several patients in both groups with abnormal liver function, likely related to surgery, anesthesia, or antibiotics rather than the study drug itself. One patient in the LB group experienced intolerable back pain, affecting pain scores and leading to follow-up discontinuation.

However, our study has several limitations. Being a single-center observational study, our results may be influenced by the specific operational and nursing standards at our center, potentially introducing bias. To minimize bias, we standardized the anesthesia protocol although variations in intraoperative pain control could arise due to different anesthesiologists. All surgeries and ICNB procedures were performed by five experienced surgeons, and variations in surgical techniques might affect postoperative pain outcomes. To ensure ICNB consistency, a dedicated team member was responsible for quality control, and blocks were considered unsuccessful if two or more intercostal spaces did not form skin wheals. Pain scores were assessed by our nursing team, and multiple training sessions were conducted to reduce potential bias. In addition, most patients had tubes removed within 48 h postoperatively, limiting the number of patients available for statistical analysis between 48 and 72 h and potentially impacting results. The sample size is also limited, which may not fully capture the diversity and complexity of a larger population. We faced a challenge with the PCIA background rate set to 0 mL/h, leading to blood return and clot formation with limb movement, increasing the risk of venous thromboembolism. To prevent clot formation, nurses used heparin saline for positive pressure sealing every morning and evening. No thrombotic complications such as venous thrombosis or pulmonary embolism occurred in this study. We hope this experience serves as a caution for future research.

## 5. Conclusions

Our center's experience indicates that LB can be safely utilized for ICNB and is effective in reducing postoperative pain in patients undergoing single-port thoracoscopic surgery, owing to its prolonged analgesic effect. LB shows great potential for inclusion in multimodal analgesia management protocols in thoracic surgery. This study employed a conservative analgesic regimen, and we plan to further investigate the efficacy and safety of using LB alone for ICNB in future thoracic surgical procedures.

## Figures and Tables

**Table 1:** The demographic and clinical baseline characteristics of the patients.

Characteristics	Control (*n* = 57)	LB (*n* = 56)	*p*
Female	36 (63.2)	34 (60.7)	0.94
Age, years, mean	57.8	58.2	0.84
Age, > 65, *n* (%)	17 (29.8)	20 (35.7)	0.64
Height, cm, mean	161.5	162.9	0.39
Weight, kg, mean	61.7	65.3	0.10
BMI, kg/m^2^, mean	23.8	25.1	0.10
Smoking	8 (14.0)	6 (10.7)	0.80
Alcohol	12 (21.1)	12 (21.4)	1
Hypertension	22 (38.6)	24 (42.9)	0.79
Pathology			0.03
Adenocarcinoma	35 (61.4)	47 (83.9)	
Squamous carcinoma	1 (1.8)	0	
Carcinoma in situ	0	1 (1.8)	
Metastasis	0	1 (1.8)	
Other malignancies	1 (1.8)	1 (1.8)	
Benign lesion	20 (35.1)	6 (10.7)	
Location of tumor			0.08
Lower left lung	5 (8.8)	12 (21.4)	
Lower right lung	13 (22.8)	5 (8.9)	
Middle right lung	8 (14.0)	3 (5.4)	
Upper left lung	16 (28.1)	16 (28.6)	
Upper left, lower left lung	0	1 (1.8)	
Upper right lung	13 (22.8)	19 (33.9)	
Upper right, middle right lung	1 (1.8)	0	
Upper right, lower right lung	1 (1.8)	0	
Operation			0.17
Lobectomy	12 (21.1)	6 (10.7)	
Segmentectomy	13 (22.8)	20 (35.7)	
Wedge resection	32 (56.1)	30 (53.6)	
Tumor size			0.71
cT1a	24 (42.1)	17 (30.4)	
cT1b	24 (42.1)	31 (55.4)	
cT1c	7 (12.3)	6 (10.7)	
cT2a	1 (1.8)	1 (1.8)	
cT2b	1 (1.8)	1 (1.8)	

*Note:* Values are *n* (%).

**Table 2:** Secondary endpoints.

Secondary endpoints	Control (*n* = 57)	LB (*n* = 56)	*p*
Remaining PCIA volume, mL	92 (86.97)	95 (90.98)	0.05
Rescue opioid usage, OME	0 (0.33.3)	0 (0.0)	0.25
Total opioid usage, OME	48 (18.93)	24 (7.5, 65.33)	0.02
Chest tube duration, h	45 (40, 61.5)	45 (41, 56.75)	0.63
Length of postoperative stay, d	3 (2.4)	3 (2.4)	0.55
OBAS	4 (3.4)	4 (4.4)	0.11
OBAS = 4	36 (63.2)	47 (83.9)	0.001

*Note:* Values are median (interquartile range) or *n* (%).

Abbreviations: LB, liposomal bupivacaine; OBAS, overall benefit of analgesia score; OME, oral morphine equivalent; PCIA: patient-controlled intravenous analgesia.

**Table 3:** Daily and cumulative postoperative PCIA usage frequency and opioid usage.

Opioid consumption, OME	Control (*n* = 41)	LB (*n* = 31)	*p*
Daily PCIA usage frequency			
0–24 h	6 (3.12.5)	2 (0.4)	0.004
24–48 h	1 (0.4)	0 (0.2)	0.06
48–72 h^a^	1 (0.3.5)	0 (0.0.25)	0.08
Daily opioid usage, OME			
0–24 h	36 (12.69)	12 (0.24)	0.008
24–48 h	6 (0.24)	0 (0.12)	0.07
48–72 h^b^	6 (0.21)	0 (0.1.5)	0.08
Cumulative PCIA usage frequency			
0–24 h	4 (1.5.10)	2 (0.4)	0.01
0–48 h	6 (3.12.5)	2 (0.4)	0.004
0–72 h	6 (3.12.5)	2 (0.4)	0.003
Cumulative opioid usage, OME			
0–24 h	36 (12.69)	12 (0.24)	0.008
0–48 h	48 (18.90)	12 (0.24)	0.005
0–72 h	54 (18.90)	12 (0.24)	0.003

*Note:* Values are median (interquartile range). Since most patients had their chest tubes and PCIA pumps removed within 48 h, only 23 patients remained using the PCIA between 48 and 72 h. 48–72 h^a^: control, *n* = 13; LB, *n* = 10; 48–72 h^b^: control, *n* = 13; LB, *n* = 10.

Abbreviations: LB, liposomal bupivacaine; OME: oral morphine equivalent; PCIA, patient-controlled intravenous analgesia.

**Table 4:** AEs occurring within 72 h postoperatively.

AE	Control (*n* = 60)	LB (*n* = 60)
Any AEs	21 (35)	15 (25)
SAE	0	0
Discontinuation due to AE	2 (3.3)^a^	1 (1.7)^b^
AEs occurring in patients		
Nausea	4 (6.7)	2 (3.3)
Vomiting	3 (5)	0
Rash	1 (1.7)	0
Dizziness	0	1 (1.7)
Palpitations	0	1 (1.7)
New-onset atrial fibrillation	2 (3.3)	0
Sinus tachycardia	1 (1.7)	3 (5)
Lower back pain	0	1 (1.7)
Abnormal liver function	11 (18.3)	7 (11.7)
Elevated amylase	4 (6.7)	3 (5)
Renal dysfunction	1 (1.7)	0
Pneumothorax after extubation	1 (1.7)	0

*Note:* Values are *n* (%).

Abbreviations: AE, adverse event; LB, liposomal bupivacaine; SAE, serious AE.

^a^One patient reported rash, and one patient reported nausea and vomiting.

^b^One patient reported severe low back pain.

## Data Availability

The data that support the findings of this study are available from the corresponding author upon reasonable request.

## Supporting Information

This file contains the patient inclusion and exclusion criteria, the standardized anesthesia protocol and surgical technique (including ICNB procedure), and the postoperative pain management strategy with PCIA settings and rescue medication details.
