# Supplementary material for: Efficacy and Safety of Bupivacaine Liposomal in Intercostal Nerve Block for Postoperative Pain Management Following Uniportal Thoracoscopy: A Randomized Trial
Source: Pain Res Manag. 2025 Jul 9;2025:8816879. doi: 10.1155/prm/8816879 (PMC12267960; doi:10.1155/prm/8816879)
Supplement: Supporting Information — Additional supporting information can be found online in the Supporting Information section. [file 8816879.f1.docx]

**Materials and Methods**

**Patients**

Inclusion criteria include an American Society of Anesthesiologists score of I-II and no history of abnormal bleeding or coagulation disorders. Exclusion criteria are: 1) Contraindications to general anesthesia or inability to undergo surgery; 2) Allergies to opioids, LB, ropivacaine, or any study medication components; 3) Absolute contraindications to NSAIDs; 4) Contraindications to ICNB and local infiltration anesthesia; 5) Long-term opioid or psychiatric medication use; 6) Recent participation in other clinical trials involving opioids; 7) Central nervous system diseases, impaired consciousness, or low educational level preventing completion of pain assessments; 8) Severe renal or hepatic impairment or other comorbidities affecting postoperative recovery; 9) BMI > 44 kg/m²; 10) Refusal to participate in the study.

Patients are excluded from the study if any of the following occur: 1) Intraoperative bleeding exceeds 500 mL or conversion to thoracotomy; 2) Complete ICNB cannot be achieved due to extensive pleural adhesions or other intraoperative issues; 3) Postoperative intensive care is required; 4) A second surgery is needed within 72 hours postoperatively; 5) AEs or patient condition deterioration; 6) Patient unblinding during the study.

**Anesthesia Protocol and Postoperative Pain Management**

**Standard Anesthesia Protocol**

Upon entering the operating room, both patient groups undergo routine monitoring, including electrocardiography, heart rate, non-invasive blood pressure, and pulse oximetry. A radial artery catheter is inserted for invasive blood pressure monitoring on the operative side. Anesthesia induction includes fentanyl (0.5 μg/kg), propofol (2-3 mg/kg), and cisatracurium (0.2-0.25 mg/kg). After achieving a bispectral index (BIS) value of 60 or lower, a double-lumen endotracheal tube is inserted for positive pressure ventilation. Anesthesia maintenance consists of 1-2% sevoflurane, propofol infusion (2-4 mg/kg/h), remifentanil infusion (0.05 μg/kg/min), and dexmedetomidine infusion (0.3-1 μg/kg/h) via intravenous pump. Intermittent doses of cisatracurium (0.1 mg/kg) are administered as needed based on surgical requirements. Anesthesia depth is maintained to keep BIS values between 40-60. Post-operatively, patients receive patient-controlled intravenous analgesia (PCIA) for pain management.

**Surgical Technique and Intercostal Nerve Block**

All incisions were consistently placed between the anterior and midaxillary lines at the 4th or 5th intercostal space, measuring 3-5 cm in length, with exact positioning and size determined by the surgical site, patient BMI, and individual anatomical characteristics.

Before closing the thoracic incision, all patients undergo thoracoscopic-guided ICNB from the second to sixth intercostal spaces. The block puncture point is located 2 cm lateral to the costovertebral junction near the intercostal vessels. A fine needle is inserted through the parietal pleura into the intercostal space, injecting 4 ml of local anesthetic between the upper and lower ribs, forming a subpleural wheal. The LB group receives LB (266 mg/20 ml), while the control group receives 20 ml of 0.375% ropivacaine. A 24 French silicone chest drain is inserted through the muscle tunnel of the original incision, with the chest tube apex positioned at the level of the second intercostal space, and layered closure is performed using absorbable sutures.

**Postoperative Pain Management and Breakthrough Pain Treatment**

Postoperative pain management for patients will utilize PCIA. The PCIA formulation includes sufentanil at a concentration of 2 μg/ml, with no background infusion, a patient-controlled bolus of 1 ml, and a lockout interval of 15 minutes. Beginning on the first postoperative day, patients will take oral celecoxib (200 mg every 12 hours) until discharge. If patients experience intolerable pain or breakthrough pain (resting visual analogue scale (VAS) score > 4), the three-step analgesic ladder will be followed. Opioid analgesics permitted are oxycodone or tramadol. For those who cannot tolerate oral medication, intramuscular injections of tramadol (100 mg) or pethidine (100 mg) are allowed. All other analgesics are prohibited within 72 hours after the administration of the study medication.
